# Analysing medical predictors for the outcome of infertility treatment: a 5-year follow-up survey

**DOI:** 10.1007/s00404-023-07097-3

**Published:** 2023-06-22

**Authors:** Lea Joana Stein, Sabine Rösner, Alessandra Lo Giudice, Beate Ditzen, Tewes Wischmann

**Affiliations:** 1 Institute of Medical Psychology, Heidelberg University Hospital, Bergheimer Str. 20, 69115 Heidelberg, Germany; 2 Department of Gynaecological Endocrinology and Fertility Disorders, Women’s Hospital of Heidelberg University, Heidelberg, Germany

**Keywords:** Assisted reproductive technology (ART), Drop-out, Infertility, Infertility treatment, Reproductive medicine

## Abstract

**Purpose:**

For many couples, bearing children is a common life goal; however it cannot always be fulfilled. Undergoing infertility treatment does not always guarantee pregnancies and live births. Couples experience miscarriages and even discontinue infertility treatment. Significant medical predictors for the outcome of infertility treatment have yet to be fully identified.

**Methods:**

To further our understanding, a cross-sectional 5-year follow-up survey was undertaken, in which 95 women and 82 men that have been treated at the Women’s Hospital of Heidelberg University participated. Binary logistic regressions, parametric and non-parametric methods were used for our sample to determine the relevance of biological (infertility diagnoses, maternal and paternal age) and lifestyle factors (smoking, drinking, over- and underweight) on the outcome of infertility treatment (clinical pregnancy, live birth, miscarriage, dropout rate). In addition, chi-square tests were used to examine differences in the outcome depending on the number of risk factors being present.

**Results:**

In the binary logistic regression models for clinical pregnancies, live births and drop outs were statistically significant only for the maternal age, whereas the maternal and paternal BMI, smoking, infertility diagnoses and infections showed no significant predicting effect on any of the outcome variables. A correlation between the number of risk factors and the outcome of infertility treatment could not be excluded.

**Conclusion:**

The results confirm that maternal age has an effect on infertility treatment, whereas the relevance of other possible medical predictors remains unclear. Further large-scale studies should be considered to increase our knowledge on their predictive power.

## What does this study add to the clinical work

**Table:** 

Significant medical predictors relating to the outcome of infertility treatment have yet to be fully identified and are the subject of investigation in this study. While the relevant influence of female age as a medical predictor on the outcome of infertility treatment can be confirmed, the significance of other possible medical predictors remains unclear.	

## Introduction

For many couples, bearing children is a common life goal. However, the desire to have children cannot always be fulfilled via spontaneous conception. These couples can consider to undergo infertility treatment [1]. Due to the influence of various individual socio-demographic, psychological and medical factors, the outcome of infertility treatment cannot always be predicted [2–4].

Previous studies primarily identify the woman’s age as a significant medical predictor for the outcome of infertility treatment [5, 6]. Older females experience significantly reduced rates of pregnancy and live births, and increased rates of miscarriage after assisted reproduction [7–9]. In addition, several studies show a positive association between older females and prematurely discontinuation of infertility treatment [10, 11]. Other possible medical predictors for the outcome of infertility treatment are biological and lifestyle factors such as infertility diagnoses, age, BMI and smoking behaviour.

In addition to the age of both partners, gender-specific causes of infertility are discussed as relevant biological factors. While female infertility can be caused by anatomical factors such as tubal occlusion, benign tumours of the uterus, uterine malformations, endometriosis or possibly cervical stenosis, as well as endocrine disorders of the hypothalamic–pituitary axis or genetic mutations, the man’s semen analysis can be impaired by testicular or ejaculatory disorders. Testicular disorders include undescended testis, varicocele, testicular trauma or torsion, hypo- or atrophy and testicular tumours. Ejaculatory disorders include retrograde or anejaculation [12]. Possible lifestyle factors that can influence the outcome of infertility treatment are dietary habits, a serious deviation from normal weight, excessive sport, nicotine and alcohol consumption, infections, the current use of certain medications, certain previous operations and, for women, the presence of insulin resistance or arterial hypertension [13, 14]. However, compared to biological factors, lifestyle factors only seem to significantly influence the outcome of infertility treatment when accumulating [13].

Although most couples with an unfulfilled desire for children show a combination of biological and lifestyle factors, these factors have not been sufficiently investigated as medical predictors for the outcome of infertility treatment in previous research. Usually, depending on the study population, only one individual factor has been deemed of significant relevance, resulting in a heterogenous general view. The main objective of this study is to identify significant medical predictors for the outcome of infertility treatment in terms of early discontinuation, the occurrence of clinical pregnancy, live birth and confirmed miscarriage. This includes confirming the predictive value of maternal age. Exploratively, other biological and lifestyle factors will be examined with regard to their predictive power. In addition, the influence of an accumulation of certain risk factors on the outcome of infertility treatment will be analysed. The results will be used to identify the relevance of medical predictors to ensure optimal individual counselling for patients with an unfulfilled desire to have children and to improve their care in fertility clinics.

## Methods

### Study design

The observational prognosis study to analyse the medical predictors for the outcome of infertility treatment is being conducted as part of the project "Follow-up survey on infertility—5 years later". In addition to the study presented here, the project consists of a master's thesis by Alessandra Lo Giudice, which deals with the psychological predictors and coping strategies for the outcome of infertility treatment [14]. The study has been registered in the German Clinical Trials Register (DRKS: 00018378).

### Data collection

Two hundred and ninety six infertile women and men who were undergoing treatment at the fertility centre of the Women’s Hospital of Heidelberg University in 2014/2015 and who had participated in the study "Coping with Infertility" by Volmer et al. [15, 16] were contacted 5 years later through a paper-based cross-sectional survey to determine relevant psychological and medical predictors for the outcome of infertility treatment. The questionnaire consists of four thematic sections. Part A collects general information such as medical details about infertility treatment and asks about an early discontinuation of treatment. Part B is aimed at couples with a still unfulfilled desire to have children and asks for information on coping mechanisms, while part C asks couples with a fulfilled desire to have children to provide information on the type of infertility treatment they received. In part D, patients can provide additional comments on the questionnaire. Ninety five women and 82 men consented and returned the completed documents. The analysis of the medical objectives are performed for couples solely, as the data of partners are assumed to be interdependent [17].

### Material

Information on the outcome of infertility treatment from the questionnaires was used for the analysis of both, psychological and medical predictors. For the medical objectives, information on the early discontinuation of infertility treatment and the occurrence of at least one live birth was taken from the questionnaires. The occurrence of at least one clinical pregnancy and at least one confirmed miscarriage was determined or confirmed through patient records from the fertility centre at the Women’s Hospital of Heidelberg University. The biological and lifestyle factors were also collected from the patient records. Sociodemographic data, such as age and previous children with each other, were taken from the initial survey by Volmer et al. [15, 16].

### Methods of analysis

Depending on the scale level, continuous and dichotomous variables were formed in order to analyse biological factors, lifestyle factors and outcomes. Depending on the distribution behaviour, parametric and non-parametric test procedures as well as chi-square tests and binary logistic stepwise backward regression models were calculated for the main objectives. The gender-specific regression models examined the predictive effect of BMI, smoking and previous illnesses of the partners on the outcome of infertility treatment. For women, age was chosen as a control variable. For the analysis of the occurrence of at least one clinical pregnancy, live birth and miscarriage, both female and male infections were also included. The relevance of the man’s infections and previous children with each other were examined as medical predictors for an early discontinuation of infertility treatment. In further analyses, chi-square tests were calculated to examine the effect of the accumulation of risk factors on the outcome of infertility treatment. For this purpose, an age > 35 years for women and ≥ 40 years for men, a deviation from normal weight with a BMI of ≥ 25 or < 18.5 kg/m^2^, a nicotine consumption of ≥ 10 cigarettes per day, a secured regular alcohol consumption of ≥ 5 drinks per week, fertility-relevant infections, current medication classified as unsafe by Embryotox or the product information, previous fertility-relevant operations and, for women, the presence of insulin resistance (tested if indicated in medical history) and arterial hypertension, were used as risk factors.

### Outcomes

**Fig. 1:**
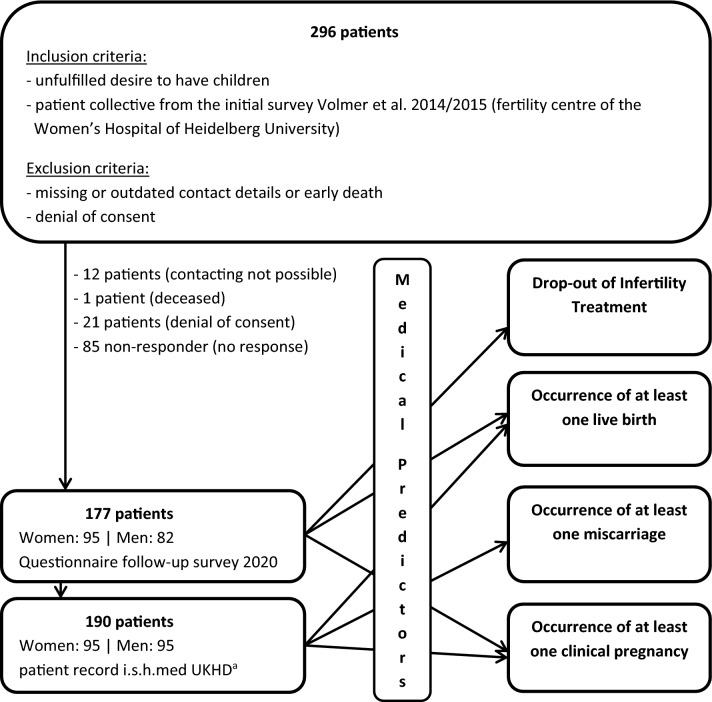
Overview of the study population and collection of endpoints (Source: own illustration). ^a^Hospital information system used by Heidelberg University Hospital

The self-reported premature discontinuation of infertility treatment, the occurrence of at least one clinical pregnancy (including spontaneous pregnancy), at least one successful pregnancy (live birth) and at least one unsuccessful pregnancy (miscarriage, stillbirth, ectopic pregnancy) were chosen as endpoints for the outcome of infertility treatment. Treatments that were discontinued without the occurrence of a pregnancy were defined as an early discontinuation of infertility treatment [18]. Clinical pregnancies were characterised by an adequate increase in hCG and typical sonographic signs [19, 20]. A successful clinical pregnancy in terms of a live birth was defined using the Personal Status Act [21]. If the criteria for a live birth were not met, a clinical pregnancy was deemed as unsuccessful, ending in a stillbirth or a miscarriage [21] (Fig. 1).

## Results

### Sociodemographic data

The average age at the beginning of the follow-up survey was 33.8 years for women and 37.03 years for men. About one third of the participants achieved an education lower than the university entrance qualification, while two-thirds completed at least the abitur or a higher educational qualification. The average partnership duration at the time of the initial study was 9.35 years, the average duration of infertility was 3.87 years and the average duration of infertility treatment was 3.81 years. Sixty three point seven percent of the participants suffered from primary sterility, while 36.3% were secondarily sterile. Paternity already existed for just under a third of the men, according to the initial survey data.

The follow-up survey identified the medical infertility diagnoses of the study population. Approximately, 39% of the cause of infertility was female-related, 15.8% male-related, 35.8% mixed, 8.4% was unexplained (idiopathic) and for 1.1% the diagnosis remained unclear.

In the present follow-up survey, 20.3% of the couples agreed to the question “We terminated a medical infertility treatment prematurely (without pregnancy or birth of a child)”. In contrast, 72.6% of couples became pregnant at least once, 69.5% gave birth to at least one child and 27.5% experienced at least one confirmed miscarriage.

### Medical predictors for the outcome of infertility treatment

The mean comparison of the age of the participants using an unpaired t-test showed a significantly higher female age (*M* = 36.88; SD = 4.83) for early discontinuation compared to continuation of infertility treatment (*M* = 33.22; SD = 4.26: *t* (21.31) = − 2.77, *p* = 0.012). For the occurrence of at least one clinical pregnancy, the woman’s age tended to be lower (*M* = 33.22; SD = 4.60) and the man’s age was significantly lower (*M* = 36; SD = 5.21) than for the absence of a clinical pregnancy (women *M* = 35.35; SD = 5.04 and men *M* = 39.77; SD = 8.33): Females *t* (41.7) = 1.88, *p* = 0.07 and males* t* (32.6) = 2.16, *p* = 0.04. For the occurrence of at least one live birth, both woman's and man's ages were significantly lower (women *M* = 33.09; SD = 4.60 and men *M* = 35.92; SD = 5.28) compared to no live birth (women *M* = 35.41; SD = 4.92 and men *M* = 39.55; SD = 7.94: women* t* (50.40) = 2.16, *p* = 0.035 and men *t* (39.33) = 2.25, *p* = 0.03). The age difference of the participants did not show a significant difference for the occurrence of at least one confirmed miscarriage. The chi-square test used to detect an association of all other investigated risk factors with the outcomes of infertility treatment only showed a significant difference in the presence of insulin resistance for the occurrence of at least one clinical pregnancy and at least one live birth, *χ*^2^(1) = 4.98, *p* = 0.03 and *χ*^2^(1) = 3.94, *p* = 0.04, respectively. Neither the smoking status, alcohol consumption, current medication, previous operations, infections of the participants nor the presence of maternal arterial hypertension showed a statistically significant association. The distribution of the gender-specific infertility causes in absolute frequencies and the chi-square-test for an association with the outcome of infertility treatment revealed a (tendential significant) difference for the occurrence of at least one clinical pregnancy for patients with endocrine disfunction (*p* = 0.062) and a significant difference for the occurrence of at least one clinical pregnancy if the woman was affected by a tubal factor (*p* = 0.04). However, only a trend could be observed for the outcome "at least one live birth" in the presence of a tubal factor (*p* = 0.09). In addition, a significant difference was shown for the same outcome in the presence of a genetic mutation in women (*p* = 0.01) and for endocrine disfunction (*p* = 0.048). The difference could only be identified as a tendency (*p* = 0.09) for the presence of a genetic mutation in the outcome "at least one confirmed miscarriage". In contrast, idiopathic infertility diagnosis, unclear diagnosis, endometriosis, hormonal imbalances and the presence of a female uterine factor did not show any statistical significance. The presence of previous paternal diseases was also statistically non-significant.

For a premature discontinuation of infertility treatment, the occurrence of at least one clinical pregnancy and at least one live birth, the binary logistic regression models showed a significant effect only for the age of the woman. With each year of age, the probability of couples dropping out of infertility treatment increases by 26.7%, while the probability of the occurrence of at least one clinical pregnancy decreases by 10.5% and the probability of the occurrence of at least one live birth decreases by 10.9%. Although the man's infections and smoking remained in the binary logistic regression models until the last step, there was no significant effect in these variables (see Table 1).

**Table 1:** Last steps of the gender-specific binary logistic regression models for all outcomes of infertility treatment

Outcomes and variables (*N* = 38–85)^a^	*ß*^b^	SE^c^	*p*	Exp(*B*)^d^	95% CI^e^	
					Lower	Upper
Drop-out						
Woman’s age	0.237	0.082	0.004**	1.267	1.080	1.487
Man’s infections	− 20.068	40,193	1.000	0.000	0.000	–
≥ 1 clinical pregnancy						
Woman’s age	− 0.111	0.055	0.043*	0.895	0.804	0.996
Man’s nicotine consumption	20.269	16,408.711	0.999	634,650,831	0.000	–
≥ 1 live birth						
Woman’s age	− 0.116	0.054	0.031*	0.891	0.802	0.989
Man’s nicotine consumption	20.392	16,408.711	0.999	717,988,819	0.000	–
≥ 1 confirmed miscarriage						
Woman’s age	0.045	0.051	0.380	1.046	0.946	1.157
Man’s infections	− 20.254	40,193	1.000	0.000	0.000	–

*p < 0.05, **p < 0.01

^a^*N* = sample size

^b^*ß* = regression coefficient

^c^SE = standard error

^d^xp(*B*) = Odds ratio

^e^CI = confidence interval

### Quantification of risk factors

About two-thirds of women and men had at least one risk factor. One third of the women and less than one quarter of the men had at least two risk factors and about 6% of women and 3% of men had at least three risk factors. Apart from a tendency towards a difference in the presence of at least one female risk factor (*p* = 0.07) and a significant difference in the presence of at least two female risk factors for an early discontinuation of infertility treatment (*χ*^2^(1) = 4.95, *p* (1-sided) = 0.01), no correlations were found between the number of risk factors of the patients and the outcome of infertility treatment (see Table 2).

**Table 2:** Cross-tabulation and chi-square-test in absolute frequencies to analyse the association between the number of risk factors and the outcomes of infertility treatment

	≥ 1 clinical pregnancy (*N* = 95)^a^			≥ 1 live birth (*N* = 95)^a^			≥ 1 miscarriage (*N* = 91)^a^			Drop-out (*N* = 79)^a^		
	No	Yes	*p*	No	Yes	*p*	No	Yes	*p*	No	Yes	*p*
Women												
No RF^b^	10	21	0.229	10	21	0.400	23	7	0.268	24	3	0.073
≥ 1 RF^b^	16	48		19	45		43	18		39	13	
< 2 RF^b^	16	48	0.229	18	46	0.233	42	19	0.132	46	7	0.013*
≥ 2 RF^b^	10	21		11	20		24	6		17	9	
< 3 RF^b^	24	65	0.368	27	62	0.439	62	23	0.370	59	15	0.494
≥ 3 RF^b^	2	4		2	4		4	2		4	1	
Men												
No RF^b^	8	23	0.406	10	21	0.400	21	9	0.353	20	7	0.183
≥ 1 RF^b^	18	46		19	45		45	16		43	9	
< 2 RF^b^	20	55	0.383	23	52	0.477	53	20	0.487	48	13	0.334
≥ 2 RF^b^	6	14		6	14		13	5		15	3	
< 3 RF^b^	25	67	0.407	28	64	0.458	63	25	0.139	61	15	0.283
≥ 3 RF^b^	1	2		1	2		3	0		2	1	

*p (one-sided) < 0.05

^a^*N* = sample size

^b^RF = risk factors

## Discussion

The delivery rate of 69.5% is quite high and corresponds to findings in other investigations [e.g. 22].

Female participants that decided to discontinue their infertility treatment showed a significantly higher age in the mean comparison. Woman’s age was also mentioned as the main reason for treatment discontinuation by 37.5% of the men, while 55.0% of the women named the treatment’s emotional burden as decisive [14]. The binary logistic regression model confirms the significance of the woman’s age. The present study confirms the result of comparative studies that identified the woman's age as a medical predictor of early discontinuation of infertility treatment [5, 6]. In addition, the comparisons of means resulted in a tendency towards a lower female age and significantly lower male age when at least one clinical pregnancy occurs. The binary logistic regression model was statistically significant for female age as a medical predictor of the occurrence of at least one clinical pregnancy. This paper confirms the result of a comparative study by Steures et al. which describes the woman’s age as a significant predictor for the occurrence of a pregnancy with the help of intrauterine inseminations [7]. The assumption of several studies that an increased male age is associated with a reduced chance of pregnancy can be confirmed in the present study too [23, 24]. The age of both partners was significantly lower in the mean comparison of the present study if at least one live birth was recorded. The present work confirms the results of comparative studies, which observe a reduced live birth rate with higher maternal or paternal age [9, 23, 24]. The assumption of the comparative studies by Yan et al. and Fossé et al. that the risk of miscarriage increases with increasing female and male age, respectively, cannot be confirmed for this sample [8, 25]. However, the relevance of the man's age should be interpreted with caution, as it is possible that its statistical significance can be explained by an association of the man's increased age with the woman's increased age [26].

The additional significant differences in the occurrence of at least one clinical pregnancy and live birth when insulin resistance is present, confirm the results of comparable studies showing lower fertility and live birth rates in women with diabetes mellitus than the control group [27, 28]. Furthermore, the chi-square test identified a significant difference for the occurrence of at least one clinical pregnancy when tubal factor was present. This association was also found in the meta-analysis by Camus et al.: The pregnancy rate for patients with tubal infertility is 20–31% depending on the additional presence of hydrosalpinx [29]. We also identified a significant difference between the presence of a genetic mutation and the occurrence of at least one live birth. The occurrence of a confirmed miscarriage tended to differ when a genetic mutation was diagnosed. In the present study, mainly hereditary thrombophilias are recorded as genetic mutations. Hereditary thrombophilias may potentially be an important cause of habitual abortion and thus prevent the occurrence of a live birth. The results of the present study are in line with the meta-analysis by Liu et al. [30]. In addition, compared with participants without endocrine disorders, participants with endocrine disorders tended to differ with respect to the occurrence of at least one clinical pregnancy and significantly differed with respect to the occurrence of at least one live birth. The present study confirms the results of the meta-analysis by Sha et al. and study by Joshi et al. which found similar and altered pregnancy and live birth rates, respectively, for patients with PCO syndrome and hyperthyroidism [32, 33]. However, an adequate comparison of the present study with other studies is difficult because of the sparse data and the lack of investigation of a pooled endocrine infertility diagnosis (rather than individual endocrine disorders). A further analysis showed a relation between the number of risk factors and the outcome of infertility treatment. Our study suggests that an increase in the number of risk factors increases the likelihood of dropping out of infertility treatment. No effect of the total number of risk factors was observed for the occurrence of at least one clinical pregnancy, live birth and miscarriage. Hassan and Killick recorded a significant effect of an accumulation of lifestyle factors on fertility [13]. This relevant effect can possibly be generalised for other outcomes of infertility treatment. However, in comparison to the study by Hassan and Killick, this association in the present study is only shown as a trend for an early discontinuation of infertility treatment.

The high response rates of 70%, inclusion of participants as couples and different rather than individual treatment methods in the study infer a good representation of the population. However, the presence of a selection bias for the initial survey is still possible due to the low response rate of about 38.5% in the original study population of 2014/2015 [15, 16]. The responder–non-responder analysis conducted as part of the master's thesis on psychological predictors and coping strategies for the outcome of infertility treatment showed no non-response bias in the response behaviour of the initial survey, but it cannot be completely ruled out for the follow-up survey [14]. Biasing memory effects can be classified as low due to the double data collection by means of both the questionnaires and the patient records. However, the interpretation of the patient records was problematic, especially with regard to endometriosis. Studies that wish to investigate the predictive power of endometriosis for the outcome of infertility treatment should carefully consider their study population, as the classification systems used so far do not usually allow to draw conclusions about the relevance of endometriosis for fertility [31–33]. In addition, there was no routine questioning on patient’s physical activity and eating habits [e.g. 34]. Thus, these lifestyle factors could not be included in the analysis.

The follow-up survey can largely confirm the results of comparable studies. However, due to the sample size of 95 couples, the overall validity is severely limited and the present cross-sectional study can only establish correlations and no causal relationships. Particularly due to the sample size, further in-depth studies with large samples and more differentiated subgroups are useful to confirm the results of the present study and to improve knowledge about medical predictors for the outcome of infertility treatment. The influence of the total number of risk factors on the outcome of infertility treatment should also be investigated, as a correlation cannot be ruled out in the present study.

In general, the population should be educated about the negative influence of increased female age as well as unfavourable lifestyle factors on fertility and the outcome of infertility treatment. The identification of medical predictors can help create transparency for couples about their individual chances of success with infertility treatment. This evidence-based knowledge forms the basis for interdisciplinary medical and psychological counselling in terms of a biopsychosocial model. This results in participatory decision-making for the continuation or an early discontinuation of infertility treatment by affected couples.
